# A Patient With Athlete’s Heart Syndrome: When the Abnormal Is Normal

**DOI:** 10.7759/cureus.56143

**Published:** 2024-03-14

**Authors:** Porfirio E Diaz-Rodriguez, Victor H Molina-Lopez, Benjamin A Gonzalez Burgos, Jose Escabi

**Affiliations:** 1 Cardiology, Veterans Affairs Medical Center, San Juan, PRI; 2 Cardiology, Veterans Affairs Caribbean Healthcare System, San Juan, PRI; 3 Internal Medicine, Veterans Affairs Medical Center, San Juan, PRI

**Keywords:** conduction abnormality, vagotony, bradycardia, athlete's heart, av dissociation

## Abstract

Long-term athletic training can result in structural and conduction changes within the heart, leading to Athlete's heart syndrome (AHS). This syndrome is characterized by increased left ventricle (LV) dimensions, thickness, and mass. Dynamic exercise significantly contributes to these alterations, with sinus bradycardia being a common conduction abnormality, often accompanied by first-degree atrioventricular (AV) block. However, higher degrees of AV conduction abnormalities, such as second- and third-degree blocks, though rare, might occur due to parasympathetic hypertonia. Prompt evaluation is necessary to rule out underlying structural or infiltrative heart diseases. We present the case of a 66-year-old lifelong long-distance runner with marked sinus bradycardia, AV dissociation, and junctional escape rhythm, alongside left ventricular hypertrophy (LVH) and T-wave repolarization abnormalities. Subsequent studies ruled out possible pathologies, and the patient was diagnosed with AHS, characterized by cardiac remodeling and bradycardia due to prolonged cardiac loading. This case underscores the importance of clinical assessment, cardiac imaging, and exclusion of pathologic causes to distinguish normal physiological adaptations from potentially concerning conditions.

## Introduction

Athletes strive for top performance in competition, while exercisers prioritize incorporating physical activity for overall health and fitness. Therefore, distinguishing Athlete's heart syndrome (AHS) from other pathological heart conditions is important [1]. Long-term athletic training has been linked to increased dimensions, thickness, and mass of the left ventricle (LV), leading to AHS [2]. This syndrome is characterized by structural and conduction changes to the heart primarily resulting from dynamic exercise. A common conduction abnormality in this population is sinus bradycardia, often coinciding with a first-degree atrioventricular (AV) block. However, higher degrees of AV conduction abnormalities may also be due to parasympathetic hypertonia [2,3]. When such abnormalities are detected, they should elicit further evaluation to exclude possible underlying pathologies, such as structural or infiltrative heart diseases, underscoring the complex interplay between intensive athletic training and cardiovascular health.

## Case presentation

**Figure 1:**
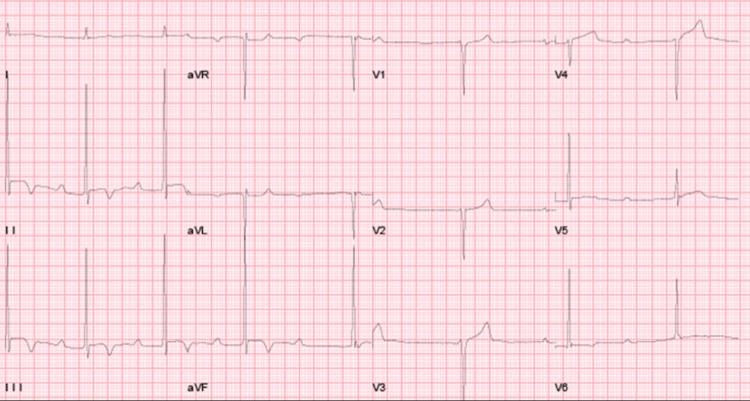
Resting ECG showing sinus bradycardia with AV dissociation and junctional escape rhythm, LVH with repolarization changes ECG, electrocardiogram; AV, atrioventricular; LVH, left ventricular hypertrophy

We present a case of a 66-year-old lifelong long-distance runner (runs an average of 55-60 miles a week; for more than 40 years) with arterial hypertension (well controlled with ranges of 105-115/60-70 mmHg), incidentally found to have marked sinus bradycardia, AV dissociation, junctional escape rhythm, normal axis (~80 degrees), and left ventricular hypertrophy (LVH) with inferolateral T-wave repolarization abnormalities (Figure 1).

**Figure 2:**
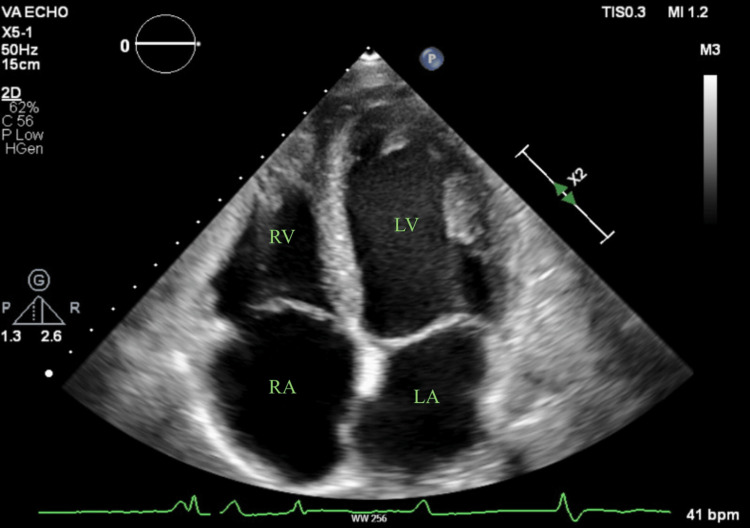
Apical four-chamber view noting biatrial enlargement, bright myocardium, and dilated LV RA, right atrium; RV, right ventricle; LA, left atrium; LV, left ventricle

**Figure 3:**
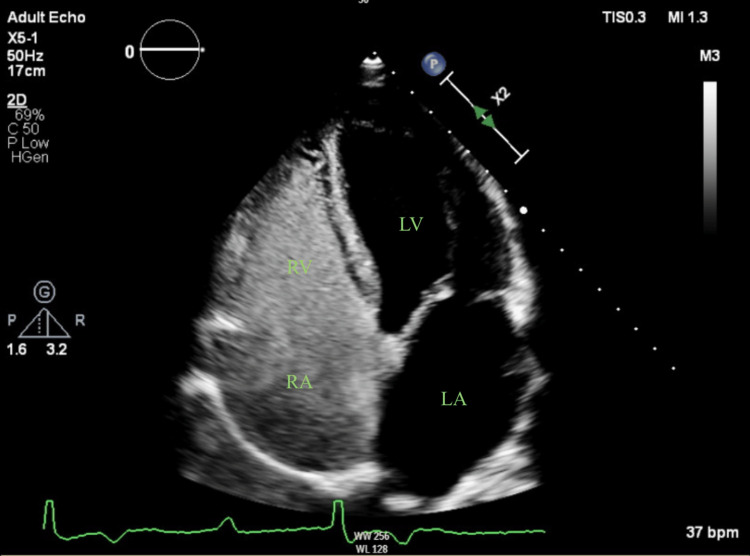
Apical four-chamber view after agitated saline injection showing no shunts RA, right atrium; RV, right ventricle; LA, left atrium; LV, left ventricle

Initial suspicion of an amyloid infiltrative disorder arose due to significant concentric LVH (IVSd: 1 cm, LVIDd: 4.8cm, and PWTd: 1.3cm, ejection fraction: 55-60%) and biatrial enlargement discovered on transthoracic echocardiogram (TTE) (Figure 2). Agitate saline was used during the TTE to rule out intracardiac shunts, which was negative (Figure 3). A comprehensive evaluation for amyloidosis, including a pyrophosphate study, serum and urine protein electrophoresis (SPEP/UPEP), a fat pad biopsy, and an ischemia evaluation due to inferolateral T-wave inversions, yielded negative results. Cardiac magnetic resonance imaging, which can be helpful in elucidating early infiltrative changes and scar tissue, was unfortunately not possible due to limited availability.

**Figure 4:**
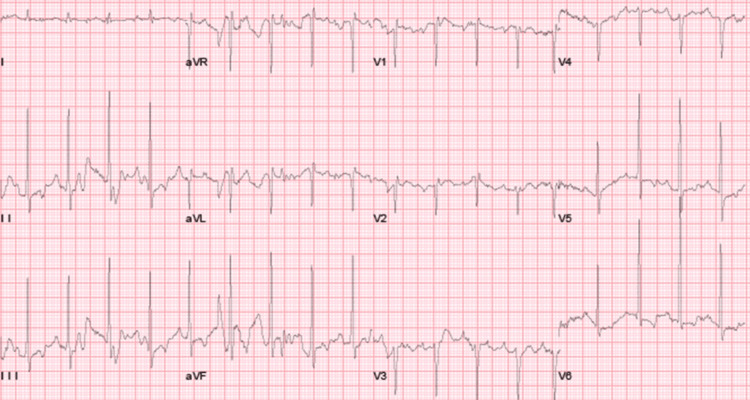
ECG at stage three of Bruce protocol with 1:1 AV conduction ECG, electrocardiogram; AV, atrioventricular

A graded stress test utilizing the Bruce protocol was performed in light of the patient's history of high-intensity lifelong training to evaluate chronotropic response [4]. It showed an appropriate chronotropic activity with a sinus rate exceeding the target heart rate and resolution of AV dissociation with a normalized 1:1 AV conduction (Figure 4). Given these findings and negative results for potential pathological conditions, the patient was eventually diagnosed with isorhythmic dissociation due to AHS, a physiological adaptation commonly seen in athletes.

## Discussion

Athletes are often presented with a range of electrocardiographic changes considered within the typical spectrum for this population (Table 1). Our patient's history of lifelong intense training in conjunction with inferolateral T-wave inversion necessitated an evaluation for potential structural heart diseases [5]. Despite the initial suspicion of amyloidosis, this was deemed less likely in this case, given the lack of characteristic low voltage QRS on ECG, only mild LVH (13 mm), and normal left ventricular (LV) diastolic function, with pyrophosphate study, SPEP/UPEP, a fat pad biopsy, all negative. Other infiltrative diseases like hemochromatosis and glycogen storage disease (GSD) were less likely to be considered in the patient's differential diagnosis due to the absence of characteristic findings such as skin changes and liver disease, as well as the absence of associated conditions like diabetes, arthropathy, and hypogonadism [6]. GSDs are uncommon congenital disorders associated with carbohydrate metabolism, typically inherited in an autosomal recessive manner [7]. Instead, the patient's presentation was more consistent with AHS, a physiological adaptation characterized by heart enlargement and bradycardia that result from repetitive cardiac loading [8-10]. Interestingly, AHS can lead to an uncommon cardiac rhythm known as isorhythmic AV dissociation, particularly in cases of extreme bradycardia. There are four mechanisms of AV dissociation: AV block, default, usurpation, and a combination of the previous mechanisms [11]. The most commonly known is AV block in which there is a disturbance in impulse conduction from the atria to the ventricles, which results in more P-waves than QRS complexes. Usurpation or acceleration happens when an abnormally enhanced discharge rate of a usually slower subsidiary pacemaker (such as ventricular tachycardia) surpasses the primary pacemaker. The mechanism of default, as in our patient, is secondary to marked slowing of the primary pacemaker (sinoatrial node) that allows the escape of a subsidiary pacemaker such as junctional and sometimes ventricular escape rhythms. Finally, a combination of the previously mentioned mechanisms can occur, most commonly seen when the intoxication of digoxin may cause AV block in combination with an accelerated junctional rhythm [11]. When the mechanism of AV dissociation is caused by default, it typically resolves as the heart rate increases above the pace of the junctional escape rhythm [11]. Given that our patient remained asymptomatic and demonstrated a normal chronotropic response to exercise, this situation was considered benign and a natural consequence of the patient's athletic conditioning. There was no indication for further invasive management, such as permanent pacemaker implantation. This case underscores the importance of careful interpretation of electrocardiograms in athletes and the need for thorough diagnostic evaluation before considering any invasive procedures.

**Table 1:** Normal EKG findings in athletes AV, atrioventricular; EKG, electrocardiogram; RBBB, right bundle branch block Sharma S, Drezner J, Baggish A, et al. International Recommendations for Electrocardiographic Interpretation in Athletes. J Am Coll Cardiol. 2017 Feb, 69 (8): 1057-1075. https://doi.org/10.1016/j.jacc.2017.01.015

Normal EKG finding	Definition
Increased QRS voltage	Isolated QRS voltage criteria for left (SV1 + RV5 or RV6 >3.5 mV) or right ventricular hypertrophy (RV1 + SV5 or SV6 >1.1 mV)
Incomplete RBBB	rSR' pattern in lead V1 and a QRS pattern in lead V6 with QRS duration <120 ms
Early repolarization	J-point elevation, ST-segment elevation, J-waves, or terminal QRS slurring in the inferior and/or lateral leads
Black athlete repolarization variant	J-point elevation and convex ("domed") ST-segment elevation followed by T-wave inversions in leads V1-V4 in black athletes
Juvenile T-wave pattern	T-wave inversion V1-V3 in athletes aged <16 y
Sinus bradycardia	≥30 beats per minute
Sinus arrhythmia	Heart rate variation with respiration: rate increases during inspiration and decreases during expiration
Ectopic atrial rhythm	P-waves are a different morphology compared with the sinus P-wave, such as a negative P-waves in the inferior leads ("low atrial rhythm")
Junctional escape rhythm	QRS rate is faster than the resting P-wave or sinus rate and typically <100 beats/min with narrow QRS complex unless the baseline QRS is conducted with aberrancy
1st degree AV block	PR interval: 200-400 ms
2nd degree AV block - Mobitz 1 or Wenckebach	PR interval progressively lengthens until there is a non-conducted P-wave with no QRS complex; the first Puerto Rico interval after the dropped beat is shorter than the last conducted PR interval

## Conclusions

In the context of a history involving vigorous, dynamic exercise that necessitates both strength and endurance, the presence of an AV conduction abnormality and corresponding structural modifications identified by 2D echocardiography should raise the index of suspicion for AHS. Nonetheless, exploring potential alternative causes beyond AHS is prudent and advisable, primarily when a patient's electrocardiographic and imaging findings lie within an ambiguous interpretive area. There is no current justification for implementing permanent pacing for patients who maintain an asymptomatic status and demonstrate an appropriate chronotropic response.
